# Is APACHE II a useful tool for clinical research?

**DOI:** 10.5935/0103-507X.20170046

**Published:** 2017

**Authors:** Rui P. Moreno, Antonio Paulo Nassar Jr.

**Affiliations:** 1 Hospital de São José, Centro Hospitalar de Lisboa Central - Lisboa, Portugal.; 2 Intensive Care Unit, A.C. Camargo Cancer Center - São Paulo (SP), Brazil.

The population of patients admitted to the intensive care unit (ICU) is quite heterogeneous. Overall, the outcome of ICU treatment depends on the site, cause of admission, age, prior comorbidities, and acute physiological changes at admission and during the first several hours of treatment. Predictions of the in-hospital mortality of ICU patients play important roles with respect to inclusion/exclusion criteria in clinical trials, comparisons of observed mortality with predicted mortality using a score, and estimations of standardized mortality ratios in populations of critical patients. The need for such predictions has led many researchers to develop equations to calculate probabilities of associated mortality. Although prognostic scores have been used since the 1950s (such as the Apgar^(1)^ score for newborns, which was developed by Virginia Apgar), their use for critically ill patients was established only in 1985, when Knaus et al. published the second version of the Acute Physiology and Chronic Health Evaluation (APACHE II),^(2)^ which quickly became the most widely used prognostic index in ICUs and clinical trials worldwide. The original description of APACHE II is the most cited study in the intensive medicine literature to date.^(3)^

The ability of a prognostic index to predict an outcome (in this case, in-hospital mortality) is assessed based on its calibration and discrimination. Calibration refers to the correspondence between expected mortality predicted using the index and observed mortality in the examined population. Typically, calibration is evaluated by comparing observed and predicted mortality in given predicted risk groups (e.g., deciles, which are used in the Hosmer-Lemeshow test).^(4)^ The calibration of a prognostic model generally deteriorates over time due to changes in ICU admission and discharge criteria, the evolution of support, and variations in the availability and effectiveness of different treatments for particular conditions. Thus, technological and scientific developments in intensive medicine over the last 30 years have rendered APACHE II obsolete. At present, this model generally overestimates mortality in many scenarios in which it is applied. Subsequent versions of this model, such as the most recent variant, APACHE IV,^(5)^ correct this problem, at least in part. As described by Soares et al.,^(6)^ APACHE II should not be used as a benchmarking tool in the ICU because almost any ICU today would be considered "high performance" based on having hospital mortality much lower than that expected in 1985.

In contrast, discrimination refers to the ability of a prognostic index to differentiate between patients who survive and patients who die. This metric is evaluated based on the area under a receiver operating characteristic (ROC) curve,^(7)^ with a larger area indicative of greater accuracy (provided, of course, that the area is greater than 0.5, the value at which discrimination is no better than chance) (Table 1).

**Table 1:** Discrimination capacity of a prognostic index based on the area under the corresponding receiver operating characteristic curve

Discrimination	Area under the curve
Excellent	0.90 - 0.99
Very good	0.80 - 0.89
Good	0.70 - 0.79
Moderate	0.60 - 0.69
Poor	< 0.60

Although the calibration of APACHE II has deteriorated over time, a MEDLINE search of studies from the prior 2 years that assessed the performance of this index shows that, overall, it continues to exhibit good or very good discrimination in the various populations in which it has been evaluated (Table 2).^(8-15)^ That is, higher APACHE II scores were associated with greater hospital mortality in the examined groups of subjects.

**Table 2:** Studies from the prior 3 years that evaluated the performance of APACHE II

Study	Country	Condition	Number of patients	AUC (95% CI)
Pérez Campos et al.^(8)^	Peru	Acute pancreatitis	334	0.85 (0.77 - 0.94)
Serpa Neto et al.^(9)^	Brazil	General ICU	3,333	0.80 (0.77 - 0.83)
Que et al.^(10)^	Switzerland	Severe sepsis/septic shock	Development (Switzerland): 158	0.64 (0.54 - 0.73)
	Brazil		Validation (Brazil): 91	0.64 (0.52 - 0.75)
Ariyaratnam et al.^(11)^	United Kingdom	Post-operative care for cardiac surgery	1,646	0.65 (0.56 - 0.74)
Williams et al.^(12)^	Australia	Admission from the emergency room for suspected infection	8,871	0.90 (0.88 - 0.91)
Hashmi et al.^(13)^	Pakistan	General ICU	213	0.83 (0.77 - 0.88)
Khwannimit et al.^(14)^	Thailand	Sepsis	913	0.91 (0.89 - 0.93)
Huang et al.^(15)^	China	Severe ARDS on ECMO	23	0.76 (0.56 - 0.96)

AUC - area under the curve; 95% CI - 95% confidence interval; ICU - intensive care unit; ARDS - acute respiratory distress syndrome; ECMO - extracorporeal membrane oxygenation.

In addition to the heterogeneity of the patient population admitted to the ICU, another consideration is that intensive medicine encompasses syndromes with equally broad spectra of presentation, such as sepsis, acute respiratory distress syndrome, delirium, and postoperative care for major surgeries. Thus, a method to measure the severity of all of these patients is required. This need is especially apparent in clinical studies, which must include a representative population sample to ensure that their findings can be extrapolated to clinical practice. APACHE II was the first index to indicate or contraindicate the use of a certain therapy (in particular, activated protein C in sepsis);^(16)^ the treatment in question was eventually determined to be inappropriate and detrimental.^(17)^ Another therapeutic intervention, the use of low doses of corticosteroids in sepsis, proved beneficial in a study that included patients with greater severity (in this case, another index was used: the Simplified Acute Physiology Score - SAPS- II)^(18)^ but not in another investigation that involved less severely ill patients.^(19)^ This difference in findings led the Sepsis Surviving Campaign to recommend the use of hydrocortisone as an option for septic shock patients who remain unstable after volume expansion and vasopressor use.^(20)^

**Figure 1:**
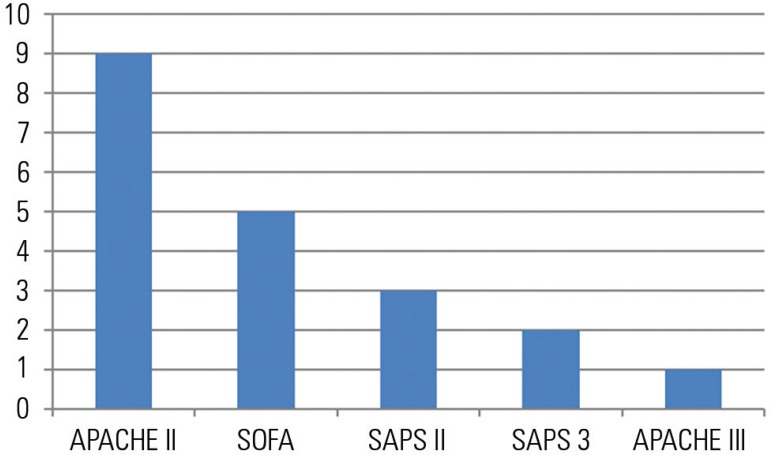
Numbers of trials in which various prognostic indices were used to describe patient severity, out of 12 clinical trials performed in intensive care units and published in the New England Journal of Medicine, The Lancet, or JAMA in 2016.^(21-32)^ These numbers sum to more than 12 because certain studies involved the use of multiple prognostic indices. APACHE - Acute Physiology and Chronic Health Evaluation; SOFA - Sequential Organ Failure Assessment; SAPS - Simplified Acute Physiology Score.

Because it continues to exhibit good discrimination capacity, APACHE II remains a widely used index to describe severity in populations of critically ill participants in clinical trials. In 2016, 12 clinical trials involving critically ill patients were published in the 3 highest-impact medical journals.^(21-32)^ APACHE II was the index that was most frequently utilized to describe the severity of the patients included in these studies; this index appeared in 9 of these 12 studies (Figure 1).

One recurring criticism of APACHE II and its subsequent versions is that these indices have been developed from an exclusively North American database. This fact introduces a large bias due to region-specific differences in the availability of different technologies^(33)^ and in patient characteristics;^(34)^ modifications to the equations used for these indices cannot fully correct for this bias.^(35)^ Today, other scores are better calibrated and should be used to assess predicted mortality^(36)^ to provide ways to express the severity of patients included in clinical trials.

However, because APACHE II continues to perform well in determining severity for a group of patients (although it cannot and should not be used to assess individual patients), its use in clinical research may be justified, in contrast to its use in the assessment of ICU performance or the prognostic evaluation of patient groups. In the latter contexts, APACHE II should return to libraries and merits respect only for having pioneered the field of prognostic evaluation in the ICU.
